# Human dental pulp stem cells modulate pro-inflammatory macrophages both through cell-to-cell contact and paracrine signaling

**DOI:** 10.3389/fimmu.2024.1440974

**Published:** 2024-10-10

**Authors:** Monia Maccaferri, Alessandra Pisciotta, Gianluca Carnevale, Carlo Salvarani, Elisa Pignatti

**Affiliations:** ^1^ Department of Surgery, Medicine Dentistry and Morphological Sciences with Interest in Transplant, Oncological and Regenerative Medicine, University of Modena and Reggio Emilia, Modena, Italy; ^2^ Rheumatology Unit, Azienda Unità Sanitaria Locale - Istituto di Ricovero e Cura a Carattere Scientifico (USL-IRCCS) di Reggio Emilia, Reggio Emilia, Italy; ^3^ Azienda Ospedaliero-Universitaria di Modena, Modena, Italy

**Keywords:** macrophages, dental pulp stem cells, inflammation, cell-culture, cell-to-cell contact, paracrine signaling

## Abstract

**Introduction:**

Macrophages play a key role in most of the inflammatory diseases such as Rheumatoid Arthritis (RA), but the mechanism underlying their pathogenesis is still under study. Among stem cells, human dental pulp stem cells (hDPSCs) have attracted attention due to their easy accessibility and immunomodulatory properties, making them a promising adjuvant therapy. In this study, we aimed to evaluate the capacity of hDPSCs to modulate the phenotypes of primary human macrophages. Additionally, we sought to observe the differences induced on macrophages when cultured directly with hDPSCs or through a cell culture insert, mimicking the paracrine communication pathway.

**Methods:**

Monocytes, isolated from buffy coats, were differentiated into pro-inflammatory M1 and anti-inflammatory M2 macrophages. Subsequently, they were cultured with hDPSCs either directly or via a cell-culture insert for 48 hours. Finally, they were analyzed for protein, gene expression, cytokines levels and immunofluorescence.

**Results:**

In our study, we have demonstrated that, hDPSCs, even without priming, can reduce TNFα levels and enhancing IL-10 release in pro-inflammatory macrophages, both through direct contact and paracrine signaling. Furthermore, we found that their effects are more pronounced when in cell-to-cell contact through the decrease of NF-kB and COX-2 expression and of CD80/PD-L1 colocalization. HDPSCs, when in contact with macrophages, showed enhanced expression of NF-kB, COX-2, ICAM-1, PD-L1, FAS-L, TNFα and IFNγ.

**Conclusion:**

We showed that hDPSCs exert immunomodulatory effects on pro-inflammatory macrophages, with cell-to-cell contact yielding a more pronounced outcome compared to paracrine signaling. Our work highlights the immunomodulatory properties of hDPSCs on activated pro-inflammatory macrophages and the potential therapeutic role in inflamed tissue.

## 1 Introduction

Macrophages play pivotal roles in both local and systemic Rheumatoid Arthritis (RA) (1, 2) and in COVID-19 hyperinflammatory syndrome (3, 4). These phagocytic cells not only eliminate microorganisms but also release proinflammatory mediators and cytokines that promote leukocyte recruitment to sites of infection (5). They also promote adaptive immune response by presenting antigens and expressing co-stimulatory molecules and cytokines to modulate specific T-cell responses (6). Conversely, they can also inhibit immune responses and promote wound healing by releasing immunoregulatory cytokines and growth factors (7). Consequently, *in vivo* macrophages exhibit a dynamic functional state primarily categorized into pro-inflammatory (M1 macrophages) and an anti-inflammatory (M2 macrophages) states depending on the microenvironment (8). *In vitro*, these states can be reproduced using cytokines such as lipopolysaccharide (LPS) and interferon γ (IFNγ) to polarize macrophages towards classically M1 or interleukin (IL) -4 and IL-13 for M2 alternatively polarized macrophages (9). Mesenchymal stem cells (MSCs) are adult stem cells capable of differentiating into various mature cell types, including osteoblasts, adipocytes, chondrocytes and reticular stroma, and possessing self-renewal properties (10). MSCs have been implicated in both in innate and adaptive immunity, exerting immunomodulatory functions through both cell-to-cell contact and paracrine signaling (11, 12). Moreover, they have demonstrated a favorable safety profile, making them promising candidates for severe, treatment-resistant RA patients (13).

Human dental pulp stem cells (hDPSCs) are adult stem cells derived from the neural crest with the ability to differentiate into multiple cell lines and with easy accessibility (14, 15). Our research group has demonstrated that hDPSCs can modulated the production of cytokines such as TNFα, IFNγ, IL-10 and IL-17A by peripheral blood mononuclear cells (PBMCs) obtained from Coronavirus disease 2019 (COVID-19) patients (16). Studies on diabetic rats have indicated that hDPSCs can promote macrophages polarization towards anti-inflammatory phenotypes (17), with similar finding observed *in-vitro* experiments using macrophages cell lines (18–20). In this study, we aimed to evaluate the capacity of hDPSCs to modulate the phenotypes of primary human macrophages polarized *in vitro* towards three different types (steady state (M0), M1, M2) with a particular focus on the pro-inflammatory phenotype. Additionally, we sought to observe the differences induced on macrophages when cultured directly with hDPSCs or through a cell culture insert, mimicking the paracrine communication pathway.

## 2 Materials and methods

### 2.1 Cell cultures

Human DPSCs were extracted from the enclosed third molar of adults (n=3) after written informed consent in accordance with the Declaration of Helsinki as previously reported (21). Briefly, the dental pulp was digested in Minimum Essential Medium with alpha modification (α-MEM) containing 3 mg/mL type I collagenase plus 4 mg/mL dispase (all from Merck KGaA, Darmstadt, Germany) and then was filtered onto 100-μm Falcon Cell Strainers to obtain a cell suspension. Cell suspension was then plated in 25-cm^2^ culture flasks and expanded in α-MEM, 10% heat inactivated fetal bovine serum (Microtech Srl, Napoli, Italy), 1% L-glutamine solution 200 mM (Merck KGaA, Darmstadt, Germany), 1% penicillin-streptomycin solution (10,000 units penicillin and 10 mg streptomycin/mL) (Merck KGaA, Darmstadt, Germany) at 37°C and 5% CO2. Following cell expansion, human dental pulp cells underwent magnetic cell sorting, using the MACS^®^ separation kit (Miltenyi Biotec B.V. & CO. KG, Germany), against the stemness surface markers STRO-1 and c-Kit, which allow to obtain a purer stem cell population within human dental pulp. The study was performed according to the recommendations of Comitato Etico Provinciale-Azienda Ospedaliero-Universitaria di Modena (Modena, Italy), which approved the experimental protocol (Ref. Number 3299/CE; 5 September 2017). hDPSCs were further expanded and used at passages 7 to 8 in compliance with previous findings (22–24).

hDPSCs were seeded alone for 48 hours at a density of 1.5×10^4^ cells/cm^2^ in wild type (wt) medium composed by Roswell Park Memorial Institute (RPMI) 1640, 10% heat inactivated Human serum type AB (hAB) (Merck KGaA, Darmstadt, Germany), 1% L-glutamine solution 200 mM (Merck KGaA, Darmstadt, Germany), 1% penicillin-streptomycin solution (10,000 units penicillin and 10 mg streptomycin/mL) (Merck KGaA, Darmstadt, Germany) also supplemented with 20 ng/ml human Macrophage Colony Stimulating Factor (M-CSF) (Merck KGaA, Darmstadt, Germany) for M0 condition or with 100 ng/ml LPS purified from E. coli 055:B5 and 20 ng/ml IFNγ for M1 condition or with 10 ng/ml IL-4 and 10 ng/ml IL-13 for M2 condition (Merck KGaA, Darmstadt, Germany).

Macrophages were obtained as follows. Buffy coats were obtained from the Azienda Ospedaliero-Universitaria (AOU) Complex structure of Immune-transfusion of Modena. Blood products of healthy donors (n=3) were tested according to Hemotherapy Guidelines for the preparation and the use of blood component issued by law 21/10/2005 no. 219 and Decreto Ministeriale 02/11/2015 of the Italian transfusion law (25, 26). PBMCs were isolated by density-gradient centrifugation using Histopaque-1077 (Merck KGaA, Darmstadt, Germany) according to the manufacturer’s protocol. Briefly, according to Miltenyi Biotech technical support suggestions, buffy coat was diluted 1:3 with a pH 7.2 solution of phosphate buffered saline (PBS), 0.5% bovine serum albumin (Merck KGaA, Darmstadt, Germany), 2 mM Ethylenediaminetetraacetic acid (EDTA) (Merck KGaA, Darmstadt, Germany). Twenty ml of diluted buffy coat was layered over 20 ml of Histopaque and centrifuged at 400 x g for 30 min. PBMCs were collected at the interface between Histopaque and plasma and washed with 20 ml of cold PBS. The suspension was centrifuged at 300 x g for 8 min and the pellet gently resuspended in RPMI 1640 (Merck KGaA, Darmstadt, Germany), 1% L-glutamine solution 200 mM (Merck KGaA, Darmstadt, Germany), 1% penicillin-streptomycin solution (10,000 units penicillin and 10 mg streptomycin/mL) (Merck KGaA, Darmstadt, Germany) and 20 ng/ml human M-CSF (Merck KGaA, Darmstadt, Germany) without serum to favor macrophages adhesion (27). PBMCs were seeded at 2.5x10^6^ cells/ml into six-well plates for subsequent indirect co-cultures, 100 mm dishes for subsequent direct co-culture and twenty-four-well plates fulfilled with Nunc™ Thermanox™ Coverslips (Thermo Fisher Scientific Inc., USA) for immunofluorescence experiments in a humified incubator at 37 °C and 5% CO_2_. After 4 hours of incubation, nonadherent cells were removed, the wells were washed with PBS, and M0 medium: RPMI with 10% of heat inactivated Human serum type AB (hAB) (Merck KGaA, Darmstadt, Germany), 1% L-glutamine solution 200 mM, 1% penicillin-streptomycin solution (10,000 units penicillin and 10 mg streptomycin/mL) was added. PBMCs were differentiated to macrophages for 7 days with M0 medium change after 3 days.

### 2.2 Macrophages polarization and hDPSCs co-cultures

At the 8^th^ day, culture medium was removed and macrophages were maintained for 18 hours (28) in M0 medium for unstimulated macrophages (M0) or adding 100 ng/ml of LPS and 20 ng/ml of IFNγ to wild type (wt) medium: RPMI with 10% hAB serum, 1% L-glutamine solution 200 mM, 1% penicillin-streptomycin solution (10,000 units penicillin and 10 mg streptomycin/mL), for polarization toward a pro-inflammatory state (M1), or toward an anti-inflammatory state (M2) adding 10 ng/ml of IL-4 and 10 ng/ml of IL-13 (29) to wt medium.

hDPSCs were harvested and resuspended in wt, M0, M1 or M2 medium and seeded alone or above macrophages in a 1:1 ratio (28) at a density of 1.5×10^4^ cells/cm^2^ for 48 hours in the corresponding M0, M1 and M2 culture media. hDPSCs were seeded directly on macrophages and, to study the paracrine effects of hDPSCs on macrophages, they were seeded into Transwell inserts with a nominal pore size of 0.4 µm (Costar Europe Ltd).

### 2.3 Labelling of hDPSCs with fluorescent probe

hDPSCs were labelled with CellTracker Orange CMRA (Thermo Fisher Scientific Inc., USA) following manufacturer’s instructions. Briefly, when the cells reached 80% confluence, the culture medium was removed and 20 µM CellTracker working solution was added. After 45 minutes of incubation the CellTracker was removed and labeled hDPSCs were seeded on macrophages M0, M1 and M2.

### 2.4 Macrophages and hDPSCs direct co-culture immunomagnetic separation

For downstream analysis, after 48 hours of macrophages direct co-culture they were separated from hDPSCs. Cells were washed with cold PBS and detached with 0.25% Trypsin/0.1% EDTA (HiMedia Laboratories GmbH, Germany) at 37°C for 3 min. Both cell types were stained with 0.4% Trypan Blue solution (Merck KGaA, Darmstadt, Germany) and counted with a hemocytometer Neubauer counting chamber. Cells were pelleted by centrifugation at 300 x g for 5 min at room temperature (RT) and resuspended in buffer composed of PBS pH 7.2, 0.5% bovine serum albumin (BSA) and 2mM EDTA. Macrophages were than separated from hDPSCs using human anti CD14 MicroBeads (Miltenyi Biotec B.V. & CO. KG, Germany) according to the manufacturer’s protocol. Briefly, working on ice and with cold reagents, cells were incubated 15 min with CD14 microbeads in the refrigerator; than were washed with buffer, centrifuged at 300 x g for 10 min, resuspended with 500 µl of buffer. The suspension was loaded onto a MS MACS column (Miltenyi Biotec B.V. & CO. KG, Germany) which was placed in the magnetic field of a MACS separator (Miltenyi Biotec B.V. & CO. KG, Germany). The unlabeled cells (hDPSCs) and CD14^+^ cells (macrophages) were collected separately and pelleted at 300 x g for 5 min.

### 2.5 RNA extraction and real-time PCR

Samples from three independent experiments were lysed and RNA was purified with miRNeasy Tissue/Cells Advanced Mini Kit (Qiagen GmbH, Germany) following manufacturer’s instructions. Briefly, cells were lysed with buffer RLT, and genomic DNA removed with gDNA eliminator spin column. The samples were transferred to a RNeasy Mini column, and it was added isopropanol to favor RNA binding. The columns were washed with buffers and 80% ethanol to eliminate all contaminants. Total RNA was eluted with 50 µl of RNase-free water and quantified with NanoDrop™ One Microvolume UV-Vis Spectrophotometer (Thermo Fisher Scientific Inc., USA). Two hundred micrograms of total RNA were reverse transcribed into complementary DNA with Applied Biosystems™ High-Capacity cDNA Reverse Transcription Kit (Thermo Fisher Scientific Inc., USA) and SimpliAmp™ Thermal Cycler (Thermo Fisher Scientific Inc., USA) following the manufacturer’s instructions. Real-time PCR was conducted in triplicate with QuantStudio™ 3 Real-Time PCR System using PowerTrack™ SYBR Green Master Mix (Thermo Fisher Scientific Inc., USA). The comparative threshold cycle (CT) value for *RPLP0* was used to normalize loading variations. The ΔCT value was calculated by subtracting control CT values (*RPLP0*) from the corresponding experimental CT (M0, M1 or M2 alone or in co-culture). The ΔCT values were converted to fold difference compared with the control by raising two to the ΔCT. Mean values of the duplicate results of three independent experiments for each sample were used as individual data for 2^−ΔCt^ and 2^−ΔΔCt^ statistical analysis. Specific oligonucleotide primers were listed in **Table 1**; the primers not indicated by a reference were provided by OriGene (Origene Technologies, Inc., USA).

**Table 1:** Specific primers for RT-PCR.

Gene	Primers	Sequence (5’-3’)	Ref
*CD14*	Forward	GCGCTCCATGGTCGATAA	(30)
	Reverse	GCCGCTGTGTAGGAAAGAAG	
*CD80*	Forward	CACCTTGCCCTTTACGTATCT	(31)
	Reverse	CTACTTCTGTGCCCACCATATT	
*HK2*	Forward	GATTTCACCAAGCGTGGACT	(28)
	Reverse	CCACACCCACTGTCACTTTG	
*CD206*	Forward	CCATCGAGGAAGAGGTTCGG	(32)
	Reverse	GGTGGGTTACTCCTTCTGCC3	
*DC-SIGN*	Forward	GAACTGGCACGACTCCATCA	(28)
	Reverse	GTTGGGCTCTCCTCTGTTCC	
*ALOX15*	Forward	CTTAAGGACGACGCCTGGTT	(28)
	Reverse	AGTTTCCCCACCGGTACAAC	
*PD-L1*	Forward	GGTTGTGGATCCAGTCACCT	(33)
	Reverse	TTGGTGGTGGTGGTCTTACC	
*IDO*	Forward	CCTGAGGAGCTACCATCTGC	(28)
	Reverse	TCAGTGCCTCCAGTTCCTTT	
*IL-6*	Forward	AGCCACTCACCTCTTCAGAACGAA	(33)
	Reverse	AGTGCCTCTTTGCTGCTTTCACAC	
*CD90*	Forward	GAAGGTCCTCTACTTATCCGCC	
	Reverse	TGATGCCCTCACACTTGACCAG	
*HLA-DRB1*	Forward	GAGCAAGATGCTGAGTGGAGTC	
	Reverse	CTGTTGGCTGAAGTCCAGAGTG	
*FAS-L*	Forward	AAAGGAGCTGAGGAAAGTGG	
	Reverse	CATAGGTGTCTTCCCATTCCAG	
*NF-KB1*	Forward	GCAGCACTACTTCTTGACCACC	
	Reverse	TCTGCTCCTGAGCATTGACGTC	
*ICAM-1*	Forward	AGCGGCTGACGTGTGCAGTAAT	
	Reverse	TCTGAGACCTCTGGCTTCGTCA	
*COX-2*	Forward	CGGTGAAACTCTGGCTAGACAG	
	Reverse	GCAAACCGTAGATGCTCAGGGA	
*IL-10*	Forward	TCTCCGAGATGCCTTCAGCAGA	
	Reverse	TCAGACAAGGCTTGGCAACCCA	
*TNFα*	Forward	CTCTTCTGCCTGCTGCACTTTG	
	Reverse	ATGGGCTACAGGCTTGTCACTC	
*IFNγ*	Forward	TTGAAGAATTGGAAAGAGGAGAGTG	
	Reverse	AAAGGAGACAATTTGGCTCTGCATT	

### 2.6 Western blot

Cells were washed in PBS and lysed on ice with Cell Lysis Buffer II (Thermo Fisher Scientific Inc., USA), 1% protease inhibitor cocktail, 1% phosphatase inhibitor cocktail and 2% Phenylmethylsulfonyl Fluoride (Merck KGaA, Darmstadt, Germany) for 30 min by vortexing at 10-minutes intervals. Lysates were harvested, centrifuged at 13,000 x g for 10 min at 4°C and aliquoted to clean microcentrifuge tubes. Protein concentration was determined using Pierce BCA protein assay (Thermo Fisher Scientific Inc., USA) and measured at 571 nm absorbance through Multiskan FC reader (Thermo Fisher Scientific Inc., USA). Proteins were denatured in a Laemmli sample buffer (BioRad, USA) with 10% of 2-Mercaptoethanol (Thermo Fisher Scientific Inc., USA) at 100°C for 5 min and then separated by standard SDS/PAGE using 10% TGX stain free Fastcast or 12% TGX stain free Fastcast polyacrylamide gels (BioRad, USA). Proteins were transferred to nitrocellulose membrane (BioRad, USA) in a semi-dry electroblotting transfer (BioRad, USA) at constant voltage of 10 V for 30-45 min. Membranes were rinsed with Tris-buffered saline 0.1% Tween 20 (TBST) buffer and blocked at RT for 5 min with EveryBlot Blocking Buffer (BioRad, USA). Membranes were probed with primary antibodies at 4°C overnight. Primary antibodies were the following: CD80 (dilution 1:2000, TA501575, Thermofisher, USA), CD206 (dilution 1:2000, MA532498, Thermofisher, USA), HLA-DRB1 (dilution 1:400, NBP2-45316, Bio-Techne, USA), FAS-L (dilution 1:1000, sc-6237, Santa Cruz Biotechnology, USA), PD-L1 (dilution 1:1000, NBP2-15791, Bio-Techne, USA), NF-kBp65 (dilution 1:1000, 510500, Thermo Fisher Scientific Inc., USA), IDO (dilution 1:250, 711778, Thermo Fisher Scientific Inc., USA), ICAM-1 (dilution 1:250, MA5407, Thermo Fisher Scientific Inc., USA), IL-6 (dilution 1:1000, 12153, Cell Signaling Technology, USA), COX-2 (dilution 1:200, AF4198, Bio-Techne, USA). Membranes were rinsed, and then incubated with the appropriate secondary antibody HRP conjugated Goat anti-Mouse IgG (H+L) (dilution 1:5000, 31430, Thermo Fisher Scientific Inc., USA) or Goat anti-Rabbit (dilution 1:8000, A6154, Merck KGaA, Germany) at RT for 1 hour. Proteins were detected with Clarity Western ECL (BioRad, USA) and imaged using a ChemiDoc Touch Imaging System (BioRad, USA). The images were analyzed with Image Lab Software (BioRad, USA).

### 2.7 ELLA cytokines test

Supernatants were analyzed for IL-10, IL-2, IFNγ, TNFα, CXCL8/IL-8 and IL-6 by Simple Plex analysis (human SPCKE-PS-005711 cartridges including IFNγ 3rd generation, TNFa 2nd generation and IL-6 2nd generation) using the ELLA microfluidic immunoassay system, following manufacturer’s instructions (ProteinSimple, San Jose, CA). Briefly, supernatants were diluted 1:2 with sample diluent, and 50 µl of this solution was added on the ELLA cartridge. Wash buffer was added to the appropriate wells on the ELLA cartridge. Sample results measured in triplicate were reported using Simple Plex Runner v.3.7.2.0 (ProteinSimple). As expected, the CVs for ELLA within the manufacturer’s recommended range were low (%CV 0.16-16.27).

### 2.8 Immunofluorescence imaging

Cells, previously grown on Nunc™ Thermanox™ Coverslips (Thermo Fisher Scientific Inc., USA), were rinsed with PBS and fixed with 4% Formaldehyde solution for 15 min. Cells were permeabilized with 0.1% Triton X-100 in PBS and blocked with Image-iT FX Signal Enhancer (Thermo Fisher Scientific Inc., USA) for 30 min. Cells were then stained for 1 hour with the following primary and secondary antibodies: CD80 (dilution 1:66, AF-140, Bio-Techne, USA) and donkey anti-goat IgG Alexa Fluor™ 546 conjugated (dilution 1:200, A32814, Thermo Fisher Scientific Inc., USA); PD-L1 (dilution 1:100, NBP2-15791, Bio-Techne, USA) and goat anti-rabbit IgG Alexa Fluor™ 488 conjugated (dilution 1:200, MA532498, Thermofisher, USA). Cells nuclei were stained with 1 μg/ml 4’,6-Diamidino-2-Phenylindole Dihydrochloride (DAPI) in PBS for 10 min and coverslips were mounted with Fluoromount-G Mounting Medium (Thermofisher, USA). Samples were observed by a Nikon A1 confocal laser scanning microscope (Nikon Inc., USA). The confocal serial sections were processed with ImageJ software to obtain three-dimensional projections, and image rendering was performed using Adobe Photoshop Software.

### 2.9 Co-localization analysis

Colocalization analyses were conducted on the best focal Z-plan among the Z-stack acquired for each field of view for best Pearson Index calculation after background subtraction. Three different images were analyzed and data were calculated as mean ± SD. The analysis was conducted using the Colocalization module within NIS-Elements AR 3.2.

### 2.10 Statistical analysis

Data were expressed as Mean ± Standard Deviation (SD) of values obtained from three independent experiments, each of them being performed in triplicate. All the data underwent One-way ANOVA followed by Newman-Keuls or Tukey *post-hoc* tests to analyze differences among three or more experimental groups (GraphPad Prism Software version 5 Inc., San Diego, CA, USA). P values lower than 0.05 were considered statistically significant.

## 3 Results

### 3.1 M1 macrophages after hDPSCs co-culture became more flatten

**Figure 1:**
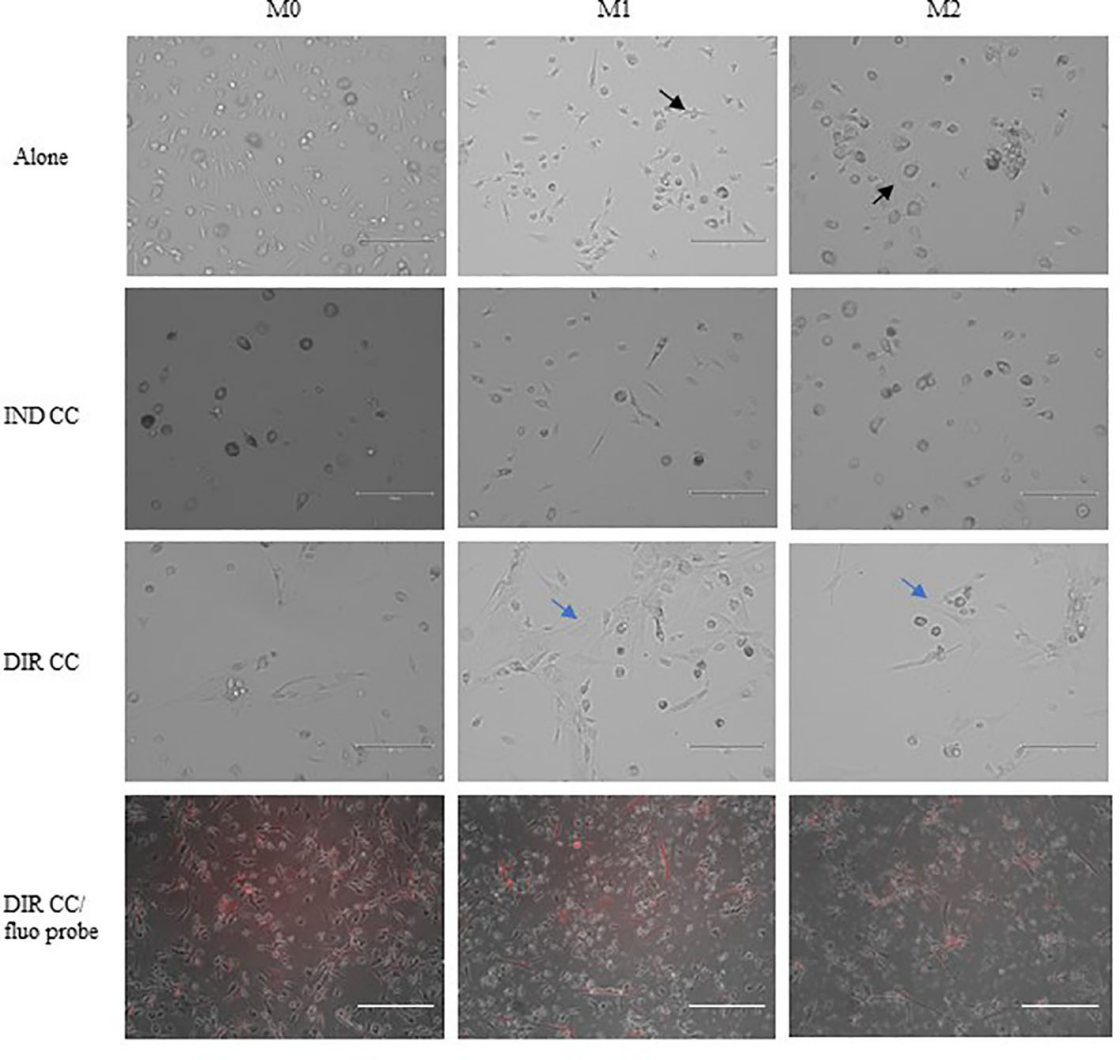
Changes in macrophages morphology. Phase contrast 20X images captured by EVOS M5000 Imaging System (Thermofisher, USA). M1 macrophages alone are irregular-shaped (black arrow) and M2 macrophages are flattened and expanded cells (black arrow). Following indirect (IND) and direct (DIR) co-culture (CC) with hDPSCs (blue arrows or in red in the images below), M1 macrophages became more rounded and M2 less expanded. Moreover, macrophages in DIR CC tend to aggregate on the top of hDPSCs. Scale bar = 150 µm.

**Figure 2:**
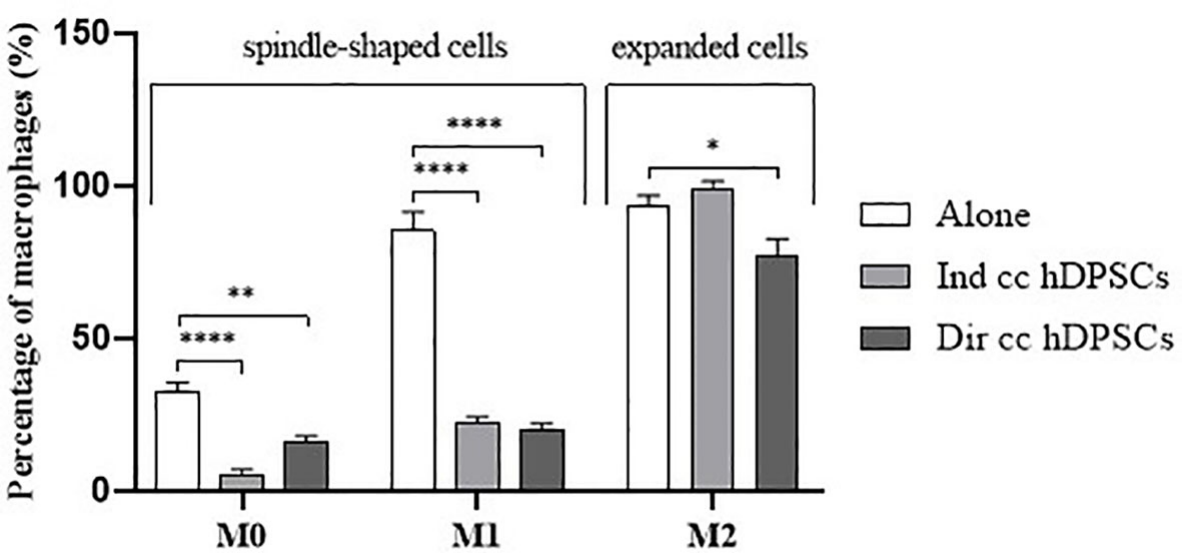
Macrophages morphology quantification. Proportion of spindle-shaped cells in M0 and M1 macrophages alone, following indirect co-culture (Ind cc hDPSCs) and direct co-culture (Dir cc hDPSCs) quantified in 20X phase contrast images, and proportion of expanded round cells in M2 macrophages alone, after indirect co-culture and direct co-culture. *p<0.05, **p<0.01, ****p<0.0001.

PBMCs were differentiated to M0 macrophages in presence of M-CSF for 7 days, then polarized to M1 and M2 alone and in co-culture with hDPSCs. M1 macrophages were mostly irregular-shaped cells, some of which were spindle-shaped. M2 macrophages were more rounded, flattened and expanded cells (**Figure 1**) as already shown by Rostam et al. (34). Following co-culture with hDPSCs, M1 macrophages tended to lose spindles and became more rounded (**Figure 1**); M2 macrophages seemed to become less expanded after direct co-culture (**Figure 1**). To quantify morphological changes, we counted spindle-shaped cells (M1) and expanded cells (M2) in three different phase contrast 20X images for each experimental condition, conducted in triplicate. Macrophages cultured alone without stimulation (M0) exhibited 32.6% ± 3.12 spindle-shaped cells. This percentage decreased to 5.3% ± 1.75 (*p*<0.0001) following indirect co-culture, and to 15.9% ± 2.03 (*p*<0.01) following direct co-culture. M1 macrophages alone exhibited 85.3% ± 6.28 spindle-shaped cells (2.6 times more respect to M0 alone). The percentage of spindle-shaped cells significantly decreased following both indirect co-cultures, resulting in 22.7% ± 1.58 (*p*<0.0001), and direct co-culture, resulting in 20.4% ± 1.71 (*p*<0.0001). M2 macrophages alone showed 93.2% ± 1.58 of expanded and rounded shape (1.4 times more respect to the expanded cells and 4.8 times less respect to spindle-shaped cells in M0 macrophages). The percentage decreased after direct co-culture, resulting in 76.7% ± 5.97 (*p*<0.05) (**Figure 2**).

### 3.2 Macrophages-hDPSCs co-culture did not affect macrophages polarization

**Figure 3:**
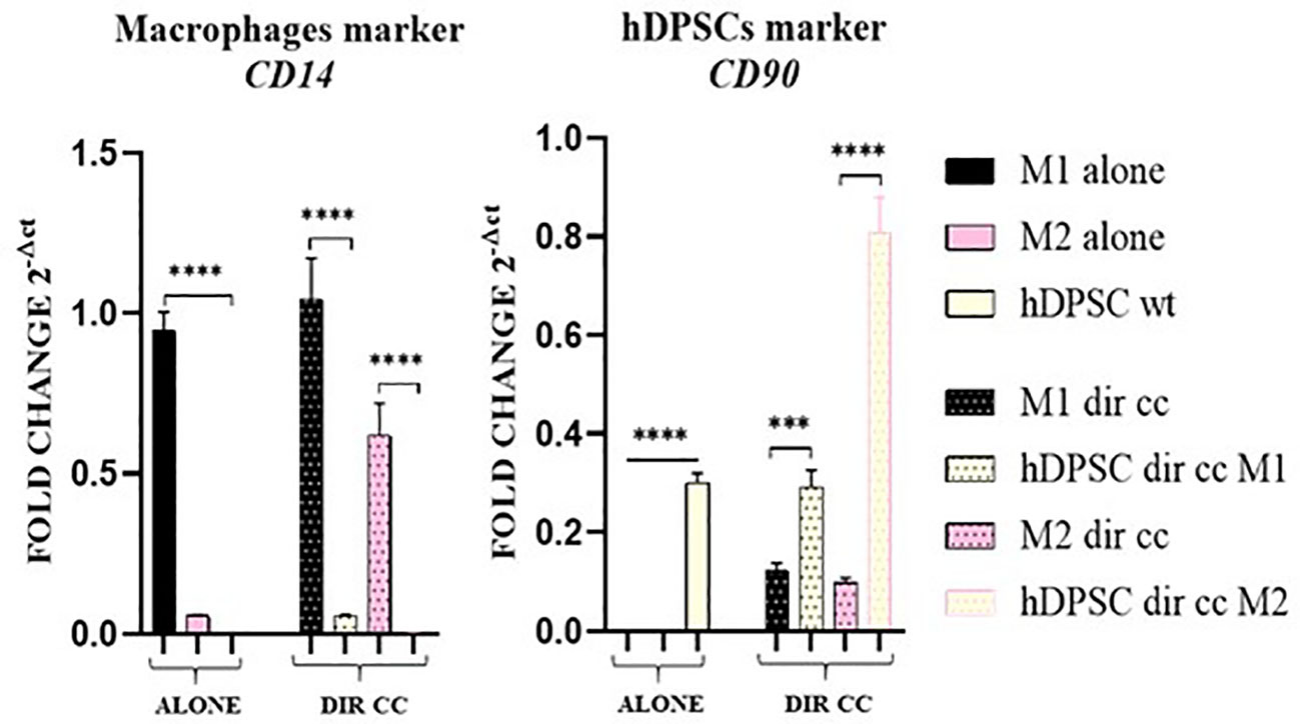
Macrophages and hDPSCs post microbeads selection. Relative mRNA expression of CD14 and CD90 normalized to RPLP0. Macrophages and hDPSCs in direct co-culture (DIR CC) were separated through CD14+ magnetic beads. The contamination of macrophages (CD14) with hDPSCs (CD90) was not significant. ***p<0.001; ****p<0.0001.

To assess the purity of the data obtained after separation of macrophages in direct co-culture with hDPSCs, we valued the expression of *CD14* (macrophages marker) and *CD90* (hDPSCs marker) genes. The contamination between the two populations was not significant (**Figure 3**).

**Figure 4:**
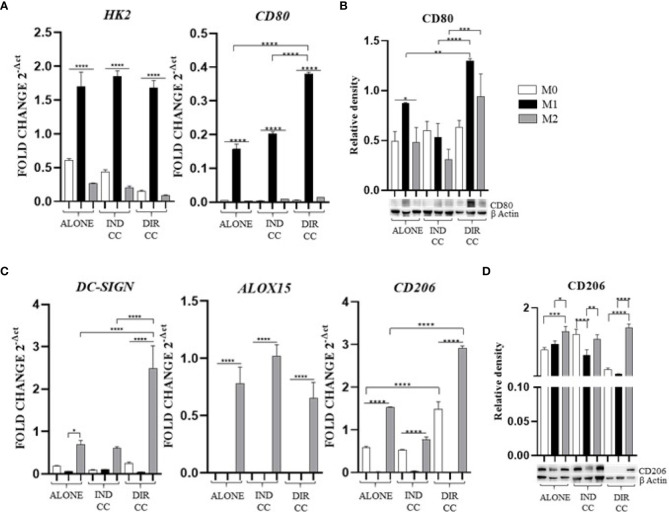
Macrophages markers expression. **(A, C)** Relative mRNA expression of M1 macrophages markers (CD80, HK2) and M2 markers (CD206, DC-SIGN, ALOX15) normalized to RPLP0. **(B, D)** Representative Western Blot depicting protein immune-probed towards CD80 and CD206; β-actin levels were used as reference to normalize data. Results from one representative experiment of three performed. Indirect co-culture (IND CC), direct co-culture (DIR CC). *p<0.05, **p<0.01, ***p<0.001, ****p<0.0001.

Polarized M1 (LPS and IFNγ stimulated) and M2 (IL-4 and IL-13 stimulated) macrophages were characterized for markers expression alone and after co-culture with hDPSCs compared to undifferentiated M0 (M-CSF stimulated) macrophages. M1 macrophages were *HK2^+^* (*p*<0.0001) and *CD80^+^* (*p*<0.0001), confirmed by RT-qPCR analysis (**Figure 4A**) and for CD80 also by protein expression (*p*<0.05) (**Figure 4B**).

Following co-culture, the polarization was kept unaffected (**Figure 4A**), except for *CD80* gene expression which was significantly higher after direct co-culture as compared to M1 alone (*p*<0.0001), data confirmed by Western Blotting analysis (**Figures 4A, B**). M2 macrophages were *DC-SIGN^+^, ALOX15^+^* (28, 35) and *CD206^+^* (**Figure 4C**)*;* for CD206 data was also confirmed by Western Blotting analysis (*p*<0.001) (**Figure 4D**). Following co-culture, the M2 polarization was kept unaffected too. The direct co-culture increased only the *CD206* (not confirmed by Western blot) and *DC-SIGN* gene expression in M2 macrophages as compared to M2 alone (*p*<0.0001) (**Figure 4C**).

### 3.3 Only hDPSCs direct co-culture decreased M1 inflammatory mediator’s expression and enhanced ICAM-1 expression

Since the pro-inflammatory stimuli administered can activate the NF-κB transcription factor and the inducible enzyme COX-2 (36), we aimed to confirm their gene and protein expression.

**Figure 5:**
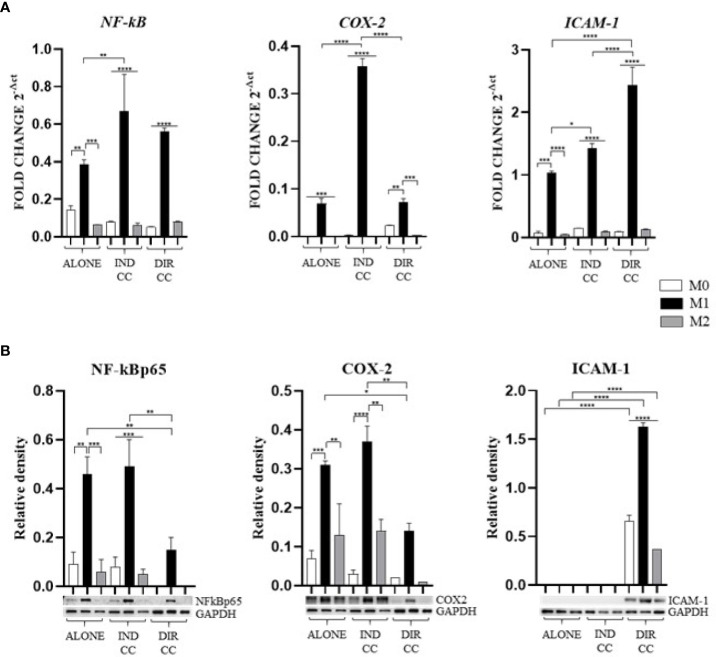
Macrophages inflammatory mediators’ markers. **(A)** Relative mRNA expression of markers related to inflammation (NF-kB, COX-2, ICAM-1) normalized towards RPLP0. **(B)** Representative Western Blot depicting proteins immune-probed towards NF-kBp65, COX-2 and ICAM-1; GAPDH levels were used as reference to normalize data. Results from one representative experiment of three performed. Indirect co-culture (IND CC), direct co-culture (DIR CC). *p<0.05, **p<0.01, ***p<0.001, ****p<0.0001.

M1 polarization was associated with the upregulation of *NF-kB* (*p*<0.01) and *COX-2* (*p*<0.001) genes, confirmed by protein expression (**Figures 5A, B**). The inflammatory environment also caused an increased expression of the adhesion related *ICAM-1* gene (*p*<0.001), data not confirmed by Western Blotting analysis (**Figures 5A, B**). Since the function of ICAM is to adhere to the endothelium to enable migration (37), it is likely that the inflammatory stimulus alone was not sufficient to induce an increase in protein expression either.

Indirect co-culture has shown to significantly enhance the expression of *NF-kB* (*p*<0.01), *COX-2* (*p*<0.0001) and *ICAM-1* (*p*<0.05) genes in M1 macrophages (**Figure 5A**) compared to M1 untreated macrophages, data not confirmed by Western Blot (**Figure 5B**).

Direct co-culture significantly decreased NF-kBp65 (*p*<0.01) and COX-2 (*p*<0.05) protein expression in M1 macrophages (**Figure 5B**), but not mRNA expression (**Figure 5A**). At opposite, it enhanced both *ICAM-1* mRNA and protein expression in all macrophage’s phenotypes (*p*<0.0001) (**Figures 5A, B**) which have been recently found to have role in resolution of inflammation (38). This allowed us to infer that cell-to-cell contact gave a stronger signal capable of reducing macrophage activation. We also observed differences between gene and protein expression which we could suppose are due to observational times and post-translational modifications (39).

### 3.4 hDPSCs regulated M1 immune modulator expression differently based on whether in direct contact or in a paracrine manner.

**Figure 6:**
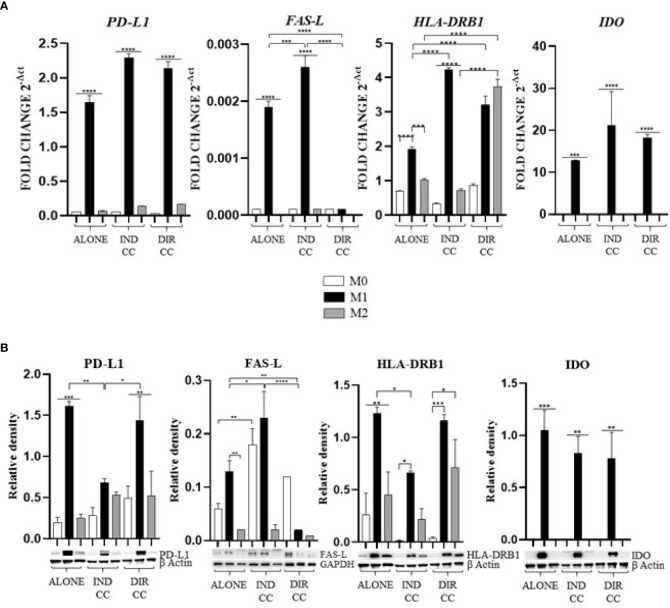
Macrophages immune modulators mediators. **(A)** Relative mRNA expression of immune regulator genes (PD-L1, FAS-L, HLA-DRB1, IDO) normalized towards RPLP0. **(B)** Representative Western Blot depicting proteins immune-probed towards PD-L1, FAS-L, HLA-DRB1 and IDO; GAPDH and β-actin levels were used as reference to normalize data. Results from one representative experiment of three performed. Indirect co-culture (IND CC), direct co-culture (DIR CC). *p<0.05, **p<0.01, ***p<0.001, ****p<0.0001.

As showed by other authors (40–43), the inflammatory medium (LPS+IFNy) caused the activation of the immune modulators *PD-L1* (*p*<0.0001), *FAS-L* (*p*<0.0001), *HLA-DRB1* (*p*<0.0001) and *IDO* (*p*<0.001) genes (**Figure 6A**). Data confirmed by the Western Blot analysis of PD-L1 (*p*<0.001), HLA-DRB1 (*p*<0.01) and IDO (*p*<0.001) proteins (**Figure 6B**).

Indirect co-culture decreased the protein expression of PD-L1 (*p*<0.01) and of HLA-DRB1 (*p*<0.05) (**Figure 6B**), but not mRNA expression (**Figure 6A**). It also increased both mRNA (*p*<0.001) and protein FAS-L expression (*p*<0.05) (**Figures 6A, B**). The expression of IDO was not affected. Soluble mediators of hDPSCs decreased the antigen presenting activity of M1 macrophages and stimulated the pro-apoptotic role by mean of FAS-L.

Direct co-culture decreased *FAS-L* mRNA and protein expression (*p*<0.0001). *PD-L1, HLA-DRB1* and *IDO* mRNA and protein expression was not affected by direct co-culture compared to M1 alone (**Figures 6A, B**).

### 3.5 Both types of hDPSCs-M1 cultures reduced TNFα and enhanced IL-10 release but also enhanced other pro-inflammatory cytokines

Cytokines level was assessed intracellularly by RT-qPCR (*IL-6, TNFα, IFNγ, IL-10*) and by Western blot (IL-6), then were measured in supernatants by ELLA microfluidic immunoassay system (IL-6, TNFα, IFNγ, CXCL8/IL-8, IL-10).

**Figure 7:**
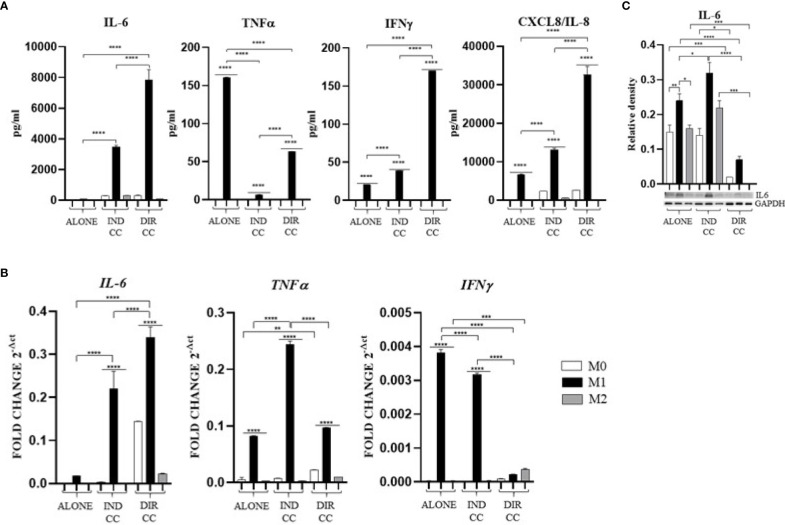
Macrophages pro-inflammatory cytokines **(A)** Cytokines levels detected in cell-culture medium by ELLA simple plex analysis. **(B)** Relative mRNA expression of pro-inflammatory cytokines genes normalized towards RPLP0 (IL-6, TNFα and IFNγ). **(C)** Representative Western Blot depicting proteins immune-probed towards IL-6; GAPDH levels were used as reference to normalize data. Results from one representative experiment of three performed. Indirect co-culture (IND CC), direct co-culture (DIR CC). *p<0.05, **p<0.01, ***p<0.001, ****p<0.0001.

Pro-inflammatory medium caused an increased cytokines level of TNFα and IFNγ (**Figure 7A**) confirmed by gene expression (*p*<0.0001) (**Figure 7B**), an increase of CXCL8/IL-8 (*p*<0.0001) (**Figure 7A**) and an increase of only IL-6 protein expression (*p*<0.01) not confirmed by gene expression (**Figure 7C**) nor by the cytokine release (**Figure 7C**). These data confirmed the activation of M1 macrophages to a pro-inflammatory status.

**Figure 8:**
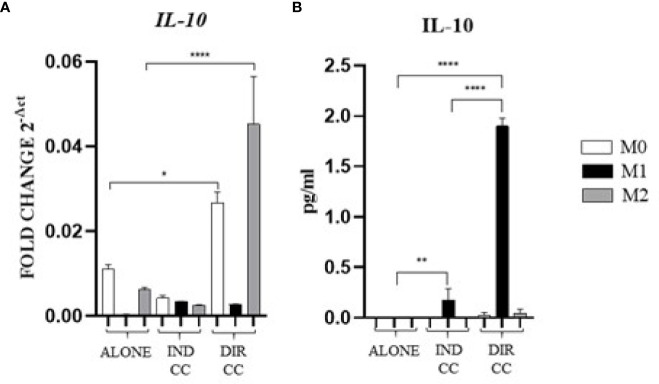
Macrophages anti-inflammatory cytokine **(A)** Relative mRNA expression of anti-inflammatory cytokine gene (IL-10) normalized towards RPLP0. **(B)** Cytokine level detected in cell-culture medium by ELLA simple plex analysis. Indirect co-culture (IND CC), direct co-culture (DIR CC). *p<0.05, **p<0.01 ****p<0.0001.

Indirect co-culture induced the increase of the pro-inflammatory CXCL8/IL-8 cytokine (p<0.0001) (**Figure 7A**) and the IL-6 cytokine (p<0.0001) confirmed by gene (p<0.0001) and protein expression (p<0.05) (**Figures 7B, C**); also, the IFNγ release (p<0.0001) was increased but in this case with a reduced gene expression (p<0.0001) (**Figures 7A, B**). On the contrary, compared to the increase in *TNFα* gene expression (p<0.0001) (**Figure 7B**), the cytokine release in the supernatant was strongly reduced (p<0.0001) (**Figure 7A**). We also observed an increased release of IL-10 (p<0.01), an anti-inflammatory cytokine (**Figure 8A**), data not confirmed by mRNA expression (**Figure 8B**).

As for indirect co-culture, the effect of the direct co-culture was to increase CXCL8/IL-8 (*p*<0.0001) cytokine release (**Figure 7A**) and IFNγ cytokine release (*p*<0.0001) with reduced gene expression (*p*<0.0001) (**Figures 7A, B**); IL-6 release increase (*p*<0.0001) was confirmed by gene expression (*p*<0.0001) but not protein expression which was reduced (*p*<0.0001) (**Figure 7C**). TNFα level in supernatant was decreased (*p*<0.0001) but not the gene expression (**Figures 7A, B**). We surprisingly found a strong increase of the anti-inflammatory cytokine IL-10 level (*p*<0.0001) (**Figure 8B**) data not confirmed by gene expression analysis (**Figure 8A**).

Considering the pro-inflammatory cytokines analyzed, hDPSCs were able to reduce only the TNFα release, but they significantly enhanced the secretion of the anti-inflammatory cytokine IL-10, especially when in cell-to-cell contact with M1 macrophages. Discrepancies were noted between mRNA and cytokines level (TNFα, IFNγ and IL-10), which could be attributed to the kinetics of cytokines processes (gene expression, transduction and release) occurring at different times. Additionally, these differences may be due to a carefully-regulated expression that involves post-translational modifications that affect mRNA stability (44).

### 3.6 CD80 PD-L1 co-localization decreased in M1 and M2 after direct hDPSC co-culture

**Figure 9:**
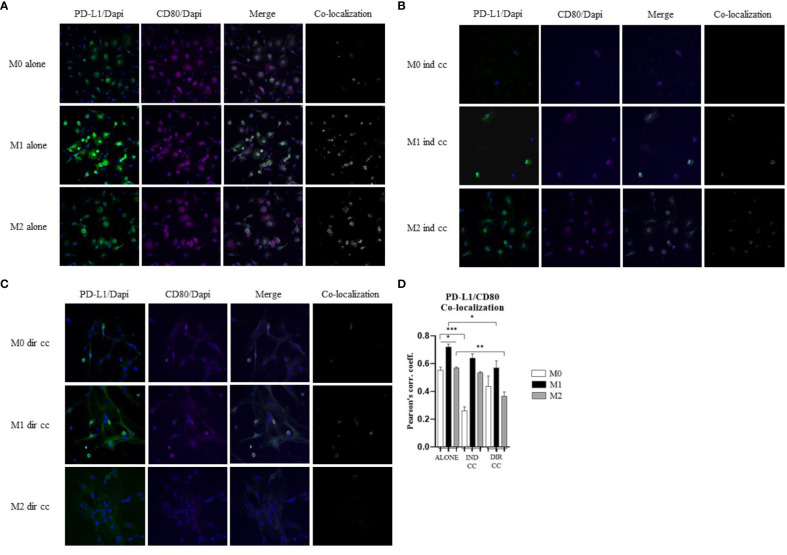
hDPSCs in direct co-culture reduce PD-L1 and CD80 colocalization in macrophages. **(A–C)** Representative images of macrophages alone, after indirect co-culture and in direct co-culture. PD-L1 was detected with rabbit-anti-PD-L1 primary antibodies and Alexa-Fluor 488-labeled anti-rabbit secondary antibodies (green). CD80 was detected with mouse-anti-CD80 primary antibodies and Alexa-Fluor 546-labeled anti-mouse secondary antibodies (magenta). Nuclei were stained with DAPI. Colocalized signals are shown in white. **(D)** Pearson correlation index between PD-L1 and CD80. (*p-value <0.05, **p-value <0.01, ***p-value <0.001).

Cells were stained with antibodies against CD80 (green) and PD-L1 (magenta) to assess the presence of protein co-localization (grey) (**Figures 9A–C**). Pearson’s correlation index was used to analyze co-localization. The results indicated that only direct co-culture significantly reduced PD-L1/CD80 co-localization in both M1 (*p*<0.05) and M2 macrophages (*p*<0.01) (**Figure 9D**). This finding suggests a potential role of hDPSCs cell-to-cell contact in diminishing PD-L1/CD80 co-localization, which has been associated with T cell activation (45).

### 3.7 Effects of co-culture with M1 macrophages on hDPSCs

To evaluate the effects exerted by medium and macrophages on hDPSCs in inflammatory conditions (LPS+IFNy), we analyzed the expression of genes mainly involved in the inflammatory response.

**Figure 10:**
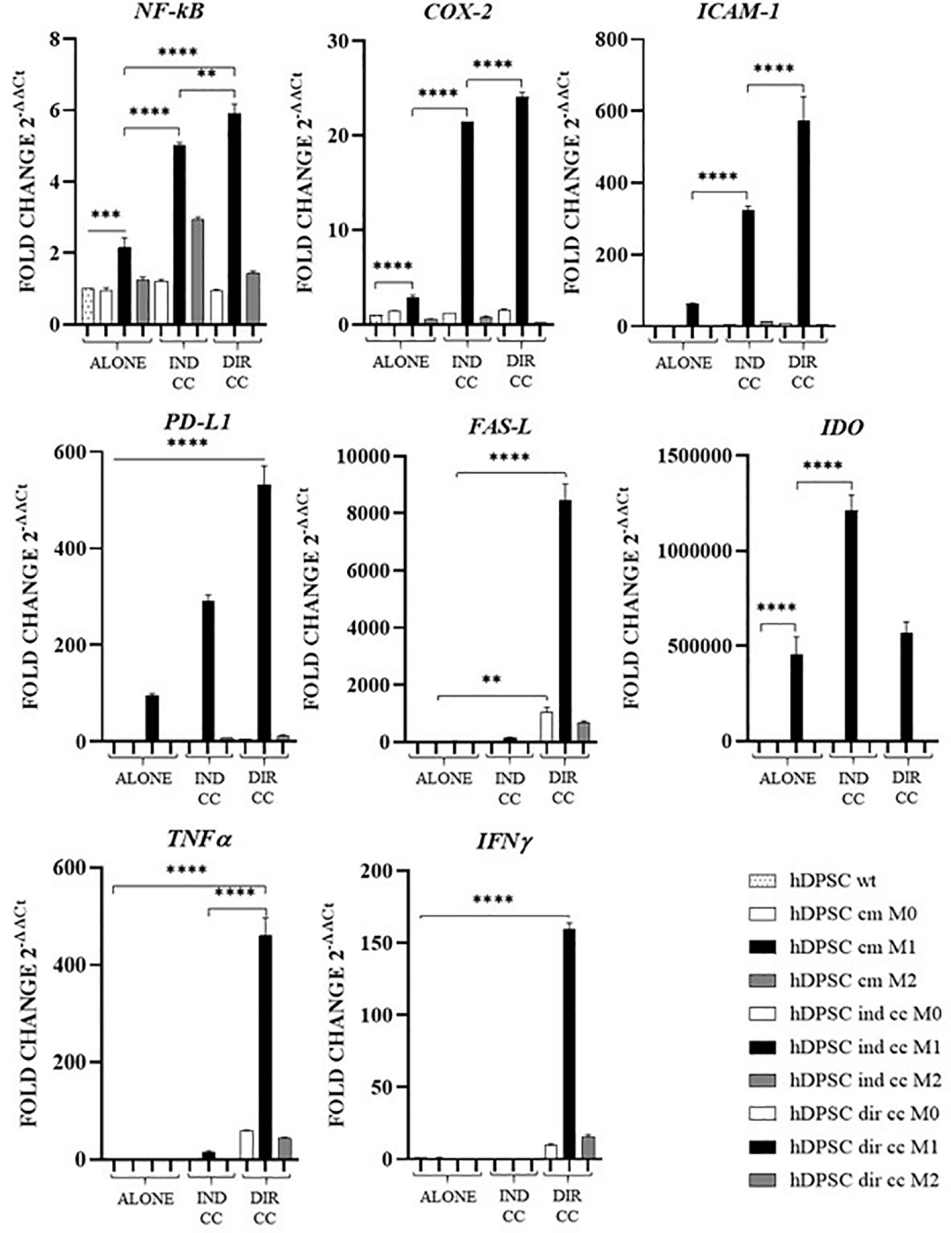
Activated genes in hDPSCs after co-culture with macrophages. Relative mRNA expression of immune regulator genes (PD-L1, FAS-L, IDO), of markers related to inflammation (NF-kB, COX-2, ICAM-1), of anti-inflammatory cytokine gene (IL-10) and pro-inflammatory cytokine genes (TNFα, IFNγ and IL-6). The gene expression of hDPSCs wt was set as 1. Results from one representative experiment of three performed. Indirect co-culture (IND CC), direct co-culture (DIR CC). *p<0.05, **p<0.01, ***p<0.001, ****p<0.0001.

The inflammatory medium caused in hDPSCs a significantly higher gene expression of *NF-kB* (*p*<0.0001) (46), *COX-2* (*p*<0.0001) (47), *PD-L1* (*p*<0.0001) (48) and *IDO* (*p*<0.0001) (49) (**Figure 10**).

Indirect co-culture with M1 macrophages increased in hDPSCs the expression of *NF-kB* (*p*<0.0001), *COX-2* (*p*<0.0001), *ICAM-1* (*p*<0.0001), *PD-L1* (*p*<0.0001) and *IDO* (*p*<0.0001) but not of *FAS-L*, *TNFα* and *IFNγ* (**Figure 10**).

Direct co-culture increased all the genes analyzed (*NF-kB, COX-2*, *ICAM-1, PD-L1, TNFα* and *IFNγ*) (*p*<0.0001) except for *IDO* (**Figure 10**).

## 4 Discussion

Our study showed that hDPSCs exert immunomodulatory effects on pro-inflammatory macrophages, with cell-to-cell contact yielding a more pronounced outcome compared to paracrine signaling. Given that approximately 30-40% of RA patients do not respond to current treatments, stem cell therapies are emerging as an alternative approach to enhance the immune response. Although the efficacy of hDPSCs has been demonstrated in various disease models, the *in vitro* mechanism underlying their properties are currently being investigated (13).

Macrophages play a key role in the pathogenesis of autoimmune diseases by promoting inflammation or fibrosis (50). It has been suggested that the activation of TLR4, a LPS receptor expressed on macrophages, may serve as a link between viral infection, inflammation and fibrosis, which are common features of interstitial lung disease (ILD) both idiopathic and associated with RA and COVID 19 (51). Additionally, the progression of RA has been strongly associated with macrophages that differentiate into pro-inflammatory phenotype, leading to the production of TNF (52). Macrophages influence T cell responses through the presence or absence of co-stimulatory receptors such as CD80/CD86 and the secretion of cytokines either by macrophages themselves or within the microenvironment (53). Recently it has been found that CD80 on APCs heterodimerizes in *cis* with PD-L1 and interacts with CD28 on T cells, thereby preventing the inhibitory effect of PD-1 (45). This interaction can activate the inflammasome and enhance the cytokines release (54). Our observations indicate that CD80/PD-L1 co-localization was more pronounced in M1 macrophages compared to M2 macrophages (**Figure 9**). Macrophages constitutively express MHC class II molecules, which are crucial for the immune response to pathogens, with higher expression levels observed in M1 macrophages due to their role in T-cells activation (8). The inflammatory state, particularly triggered by LPS and IFNγ, has been observed to induce the release of TNFα which in turn enhances the expression of NF-kB-p65 and of the inducible isoform COX-2 (36). It is also understood that following a pro-inflammatory stimulus, establishing tissue homeostasis and curbing an excessive response necessitates the activation of pro-apoptotic signals. Indeed, our differentiation of macrophages into M1 phenotype revealed heightened levels of HLA-DRB1 compared to M2 macrophages. We have further demonstrated that M1 macrophages exhibit heightened activation in response to inflammatory stimuli, characterized by elevated levels of NF-kB-p65, COX-2, TNFα, IFNγ and CXCL8/IL-8. Despite their robust response to the inflammatory environment, M1 macrophages are also activated to limit the inflammatory response through the expression of PD-L1, FAS-L and IDO.

hDPSCs have been shown to modulate macrophages function via TNFα/IDO axis and the NFκB p65 signaling pathway (18–20). This modulation occurs both indirectly (18) and through direct cell contact (17). Thus, we co-cultured M0, M1 and M2 macrophages indirectly and directly with hDPSCs. Our aim was to evaluate the effect of hDPSCs on macrophages stimulated in the M1 inflammatory phenotype and the M2 reparative phenotype, with a primary focus on the inflammatory one. Additionally, macrophages can be characterized by their morphology, with M1 macrophages predominantly exhibiting a dendritic shape, while M2 macrophages tend to have a more rounded and flattened appearance (34). Our investigation revealed that hDPSCs influenced the morphology of M1 and M2 macrophages towards a reparative phenotype, as observed through phase contrast microscopy. However, under our experimental conditions, we found that hDPSCs did not alter the phenotype of macrophages, either through direct contact or paracrine signaling. This aligns with findings by Ren et al., who studied the immunomodulatory capacity of mesenchymal stem cells (MSCs) (55). We observed that hDPSCs wild type alone did not induce any polarization of M0 macrophages unless after exposure to LPS and IFNγ. These findings are in accordance with previous research showing that preconditioning MSCs with either TLR4 agonists (56), IFNγ (57) or conditioned media from stimulated macrophages triggers the immunomodulatory potential of MSCs (58, 59). Nonetheless, M1 macrophages indirectly cultured with hDPSCs exhibited significantly lower levels of HLA-DRB1 (**Figure 6**) and TNFα (**Figure 7**), along with enhanced expression of FAS-L (**Figure 6**) and IL-10 (**Figure 8**). Furthermore, we noted an increased expression of pro-inflammatory cytokines such as IL-6, IFNγ and CXCL8/IL-8 (**Figure 7**). This observation led us to hypothesize a role of soluble factors released by hDPSCs in suppressing antigen presenting function, that we could speculate to be mediated by the IDO and NF-kB signaling pathway, which expression we found to be significantly elevated in hDPSCs (**Figure 10**) (18). However, this suppression did not occur through the NF-kB pathway in macrophages and was not sufficiently strong enough to inhibit other pro-inflammatory cytokines.

Our findings indicate that only cell-to-cell contact significantly reduced CD80/PD-L1 colocalization, as well as NF-kB and COX-2 expression in macrophages (**Figures 5**, **9**). Investigating the potential mechanism by which hDPSCs modulated M1 macrophages function, we also found that, following direct co-culture, both macrophages and hDPSCs shown significantly higher expression of ICAM-1 (**Figures 5**, **10**). ICAM-1 serves as both an adhesion molecule and signaling receptor, facilitating communication between the extracellular environment and intracellular pathways. Recent research has highlighted the role of ICAM-1 in promoting macrophages efferocytosis, which is crucial for resolving inflammation and restoring homeostasis (60). Microscopic analyses, including microscope phase contrast and confocal microscopy revealed that macrophages predominantly migrated onto the surface of the hDPSCs (**Figures 1**, **9**). The interaction between cells led to the upregulation of ICAM-1 expression, which may play a crucial role in enhancing the immunosuppressive properties of hDPSCs (61, 62). Alongside the upregulation of ICAM-1, we observed that hDPSCs exhibited significantly elevated levels of TNFα and IFNγ (**Figure 10**), with high concentrations of these cytokines also detected in the culture medium (**Figure 7A**). Previous studies have demonstrated that IFNγ is necessary for the induction of ICAM-1 and is critical for the immunosuppressive functions of MSCs (63). Therefore, we propose that ICAM-1 and IFNγ may play a role in modulating the activation of M1 macrophages by hDPSCs. Additionally, we observed that cell-to-cell contact downregulated FAS-L expression in M1 macrophages. In contrast, the expression of FAS-L and PD-L1 was upregulated in hDPSCs (**Figure 10**). Consistent with our previous findings (64), we might argue that this event is driven by the activation of immune-regulatory FAS/FAS-L pathway in hDPSCs, with further enhancement by PD-L1. Notably, IL-10 plays a pivotal role in inhibiting the inflammatory response to prevent excessive responses and restore tissue homeostasis (65). Only when M1 macrophages and hDPSCs were in direct contact, they produced IL-10 (**Figure 8**) and induced a reduction in PD-L1/CD80 co-localization in macrophages (**Figure 9**). In conjunction with the observed reduction in NF-kB and COX-2 macrophages expression, these findings suggest that hDPSCs exert a dampening effect on macrophage inflammatory response, particularly through cell-to-cell contact. The stronger effect of hDPSCs, when in contact with macrophages, we could hypothesize to be mediated by adhesion molecule ICAM-1 and IFNγ (66) activating the transcription factor NF-kB which in turn enhance the expression of COX-2 (67) and FAS-L (68).

To achieve a comprehensive understanding of the immunomodulatory response, further studies could be conducted employing various strategies. Pre-conditioning is a technique used to enhance the immunomodulatory properties of stem cells (58, 59). It could be employed to boost the immunomodulatory capabilities of the hDPSC by priming the cells to produce a higher quantity of anti-inflammatory cytokines. Further studies are necessary to identify the most effective pre-conditioning protocols and to fully understand the underlying mechanisms (69). Vasculogenic factors secreted by hDPSCs, like vascular endothelial growth factor (VEGF) (70), promote the formation of new blood vessels, which accelerates wound healing and tissue repair (71). Recently, it has been found that hyperbaric oxygen therapy can improve the vasculogenic properties of mesenchymal stem cells in inflammatory conditions *in vitro* (72). This would allow for the further assessment of hDPSCs immunoregulatory effects on macrophage polarization and function. Moreover, there is a need for further investigation into the role of PD-L1/CD80 in *cis* expression on macrophages, as this could provide valuable insights into the mechanisms underlying the immunomodulatory effects of hDPSCs. Additionally, testing hDPSCs on macrophages isolated from RA patients, is essential to understand how hDPSCs may modulate the immune response in a disease-specific context. Expanding the sample size of healthy subjects and conducting *in vivo* studies would also be valuable. While *in vitro* studies provide important insights, it is essential to validate these findings in an *in vivo* setting, as macrophages differentiated *in vitro* may not fully reflect the phenotype and behavior of macrophages found *in vivo*. Overall, these additional studies would contribute to a more comprehensive understanding of the immunomodulatory potential of hDPSCs and their potential therapeutic applications in autoimmune diseases such as RA.

This preliminary study investigating the impact of hDPSCs on macrophages from healthy subjects revealed that both indirect and direct co-culture exert immunomodulatory effects on pro-inflammatory macrophages. However, cell-to-cell contact appears to have a stronger influence, as evidenced by its role in inhibiting NF-kB and COX-2, likely mediated by ICAM-1 signaling. Moreover, cell-to-cell contact led to a decrease of CD80/PD-L1 colocalization, further supporting its immunomodulatory effects. These findings suggest that direct interaction between hDPSCs and macrophages plays a significant role in modulating macrophage function and inflammation.

## Data Availability

The original contributions presented in the study are included in the article/supplementary material. Further inquiries can be directed to the corresponding author.
